# Carcinoma of unknown primary abuts left clavicle: Case report and review of the literature

**DOI:** 10.1016/j.ijscr.2019.12.019

**Published:** 2019-12-19

**Authors:** Georgios Geropoulos, Sofoklis Mitsos, Savvas Lampridis, Martin Hayward, Marco Scarci, Nikolaos Panagiotopoulos

**Affiliations:** a Thoracic Surgery Department, University College London Hospitals, NHS Foundation Trust, London, UK; b Thoracic Surgery Department, San Geraldo Hospital, Monza, Italy

**Keywords:** Carcinoma of unknown primary, Thoracic surgery, Case report

## Abstract

**Introduction:**

Carcinoma of unknown primary is a well-recognized clinical syndrome which accounts for the 3–5% of all the malignancies. Patients with carcinoma of unknown primary usually present with late stage disease without having identified the primary source of the tumour despite an extensive diagnostic work-up.

**Presentation of case:**

A 60 years old male presented to the clinic complaining of a neck mass to the left lateral neck. Patient’s history was unremarkable without evidence of any malignant disease. Clinical and radiological examination revealed a cystic mass extending from the lower one third of the neck to the left clavicle causing periostal reaction. Mass biopsy and PET-CT was unspecific for the primary origin of the mass. However in the context of tumour immunohistochemistry, HPV status, neck location and basaloid cell differentiation, the tumour mass was considered as carcinoma of unknown primary with possible oropharyngeal primary location. The patient underwent surgical resection of the mass, left clavicle and the first rib. One year after the operation the patient is disease free.

**Discussion:**

Although CUP usually presents with cervical lyphadenopathy, in our case there was no evidence of lymph node tissue infiltration in the neck region. Surgical resection of the mass showed that the location was extending within the cervical soft tissues and upper thorax. Taking into consideration the absence of lymphadenopathy this is an uncommon location of carcinoma of unknown primary in the neck.

**Conclusion:**

This is an uncommon location of CUP with possible implications in survival and management.

## Highlights

• Carcinoma of unknown primary clinical manifestations usually associated with head and neck region.
• Painless cervical Lymphadenopathy is the most common symptom of the disease.
• Laboratory tests usually do not identify the primary disease.
• Limited data exists about management and the role of surgery in carcinoma of unknown primary if a single neck mass exists without evidence of lymph node implication.

## 1 Introduction

Carcinoma of unknown primary (CUP) is a clinical entity that accounts for about 3–5% of all malignancies. The mean age of presentation is the 75–79 years age group [1]. This condition is associated with increased rate of morbidity and mortality because already the disease is systemic with a median survival of ranging between 8 weeks and 24 months, depending on the age of presentation [2]. The majority of CUP cases are adenocarcinoma originating from infraclavicular tissues like pulmonary (most frequent), gastrointestinal tract and breast. From the supraclavicular region the most common origin is the aerodigestive tract followed by the thyroid gland. In addition, CUP metastasis in the upper two thirds of the neck usually associated with aerodigestive tumour origin instead. CUP metastasis in the lower one third neck usually has infraclavicular origin like lung adenocarcinoma [3]. As for the clinical presentation the patients usually presents with an asymptomatic lateral or less frequent middle neck swelling [4,5]. Patient’s history usually reveals smoke or alcohol consumption in older patients. Human pappilomatous virus infection is common in younger patients [6]. Nevertheless, the CUP rarely extends downward to the chest and the majority of the cases are inoperable with short life span. Purpose of this case report is to present the surgical management of a male patient with an uncommon CUP metastasis to the upper thorax without evidence of lymph node disease. This work adheres with the SCARE criteria for case reports [22].

## 2 Case report

**Fig. 1:**
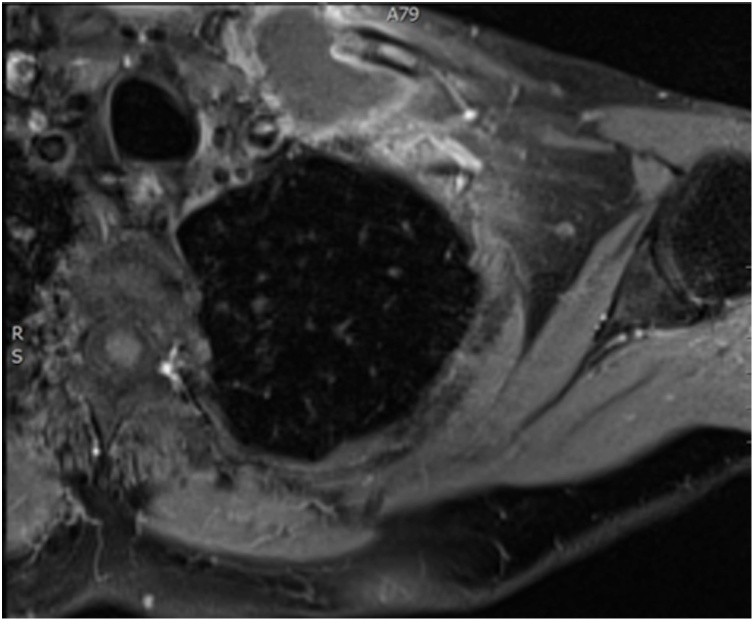
CT of thorax. There is a bilobular low-density mass with an intermediate density wall in the left side of root neck measuring approximately 6.5 × 3 cm in maximum axial dimensions and 4 cm craniocaudially. It abuts the superior aspect of clavicle extending of anterior and posterior to it. The periostal was seen sclerotic in the left clavicle in comparison to the right side. The left subclavian vein and lower left great internal jugular vein appear to be compressed by the mass.

**Fig. 2:**
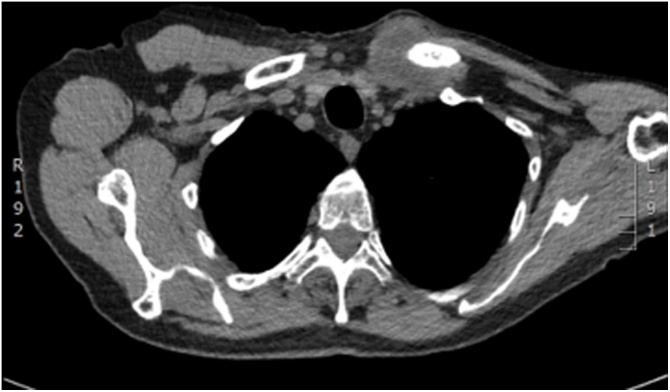
MRI of the upper thorax and brachial plexus. A cystic mass with infilterating nodular enhancing walls centred at head of clavicule, infiltrates the clavicular and sternal heads of the left sternocleidomastoid muscle, the sternohyoid muscle inferiorly and the medial end of the first rib. The clavicle is infiltrated from the head medially to the mid-third laterally over a distance of at least 6 cm. In the neck and posteriorly the left ICA is well clear of the mass, but the distal left IJV is not well seen. Subclavian vein and distal vertebral vein run immediately posterior to the mass and are inseparable from it. Subclavian artery and brachial plexus do not appear to infiltrated.

**Fig. 3:**
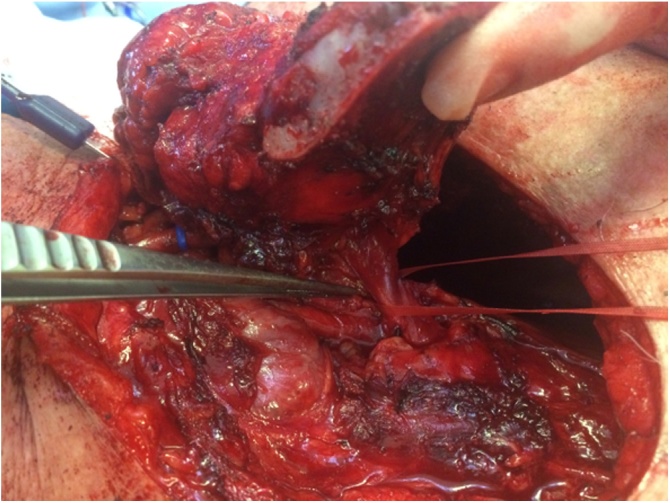
Intraoperative picture of the mass and its adhesions to the underlying first rib. The mass was totally resected with the underlying first rib and clavicle.

**Fig. 4:**
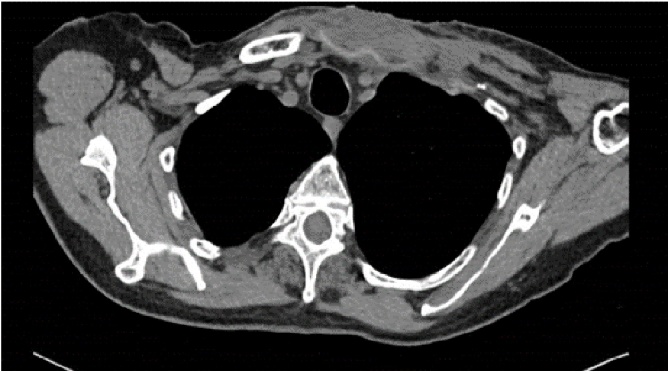
CT after the operation. There is residual, poorly defined soft tissue within the surgical bed, which is thought to represent a combination of post-surgical change and interval radiotherapy, rather than recurrent or residual tumour.

**Fig. 5:**
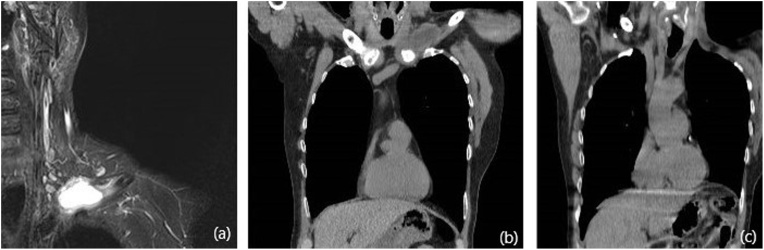
Coronal planes preoperatively in MRI (a) and CT (b) scan. Imaging one year after removal of clavicle, first rib and tumor. No evidence of disease recurrence (c).

A 60 years old male patient presented to the clinic with a primary complain of unilateral neck swelling and local pain. He first noticed this swelling about 5 weeks ago in the left lateral neck. Since then this neck lump has been getting larger rapidly and extending to his left clavicular region. Clinical examination reveals a non-tender 4 cm lesion within the left root of neck at the level V. The lesion was firm, immobile and adherent to the adjacent tissues extending downwards to the left clavicle. Past medical history was non contributary. Clinical findings are suggestive for a possible malignant lesion so the patient immediately underwent an extensive diagnostic work-up. Blood tests including biochemical and malignant biomarkers were in normal range. U/S examination reveals a large complex mixed cystic/solid mass at the root of the neck on the left side. The mass filled the supraclavicular fossa. Medially, it extended over the front of the clavicle and even on the infero-anterior aspect of the clavicle. It measured approximately 4.4 × 3.4 cm in the coronal plane and axially around 6.6 cm. These findings were confirmed by full body-CT scan, which also described the presence of the mass effect on left subclavian vein and left lower portion of the left internal jugular vein (Fig. 1). MRI depicted no infiltration of the branchial plexus (Fig. 2). In addition, there was no evidence of cervical lymphadenopathy. The PET-CT scan showed a FDG avid necrotic cervical node at the left neck. Cone U/S-guided biopsy of the lesion reveals an atypical epitheliod population, without evidence of lymph node existence in the specimen. Local immunohistochemistry was positive for p63 and CK5/6, and negative for CEA, Melan-A, PSA, CDX2, CK7, CK20, TTF1 and S100. Additional stains for CD30, OCT ¾, EBER-ISH, EMA and synaptophysin was negative and staining for p16 show strong uniform nuclear positivity. Immunohistochemistry failed to indicate the exact origin of the neoplasm. However, the combination of head and neck position, squamous cell markers (positive basaloid cell staining) and HPV surrogate p16 positivity maybe associated with a metastatic carcinoma of primary tumour which was probably located at the head and neck region (probably oropharyngeal portion). Nevertheless, multiple biopsies from the oropharyngeal region including tongue, tonsils and nasal cavity were normal. Furthermore the patient underwent tonsillectomy and the microscopical examination of the specimen was normal. Surgical excision of the mass was decided. An anterior incision according to the dartevelle approach was performed (Fig. 3). This approach gave us the opportunity to carefully dissect the neck and thoracic portion of the tumour. By the end of the operation the gap occurring by the successfully complete resection of the mass, left clavicle and first rib was covered by a vascular muscle flap from the major pectoralis muscle. Microscopic examination of the mass surrounding the clavicle showed nests of epithelial cells with pseudoglandular structures and evidence of focal keratinisation together with central necrosis. There was perineural invasion without evidence of lympovascular tumor infiltration.The clavicle bone fragment showed no abnormality and cervical lymph nodes IV and V were negative for malignant disease. The patient after 7 days discharged without any major postoperative complication and scheduled to radiotherapy. However in the context of the possible muscle flap necrosis the radiation therapy was postoponed until the viability was confirmed. Follow-up of the patient after one year showed no evidence of metastastic disease or disease progression (Figs. 4 and 5).

## 3 Discussion

Patients with biopsy proved CUP should follow an extensive work up including CT, PET-CT, Upper and Lower GI tract endoscopy, Immunohistochemistry (IHC) and anything else with the potential to diagnose the primary location of the malignancy [7]. Deonarine et al. in their retrospective study concludes that 18FDG PET-CT was able to detect the primary of CUP with 74.5% accuracy. Nevertheless, in most cases the definite diagnosis usually is given by the immunohistochemistry [8]. The development of mononuclear antibodies against several human cells antigen gave us the opportunity to approximate the origin of the neoplastic cells. Tomuleasa et al. in their study gave the example of the most common IHC staining: CK7 and CK20. The CK20 stains positive for tumours originating from the gastrointestinal tract and its accessory organs. While, CK20 stains positive for lung, thyroid and gynaecological tumours [7]. However this is a trace paradigm of how the IHC can distinguish the origin. Recent studies indicate that the combination of IHC with HPV status and molecular technics (mRNA, miRNAs, DNA or epigenetics) significantly raises the chance of identifying the primary tumour source, predict outcome and guide the appropriate treatment [9,10]. The cystic appearance of our case is a possible predictor of HPV infection and gave us a clue about the possible location of the primary [11,12]. Yasui et al. in their study concludes to a strong correlation between cystic appearances of CUP and HPV [13]. El-Mofty et al. studied the incidence of HPV infection in 23 cases of HPV positive CUP: 22/23 the location of primary tumour was along the oropharyngeal tract with 95.7% sensitivity and 85.7% specificity [11]. The optimal treatment for CUP is surgical removal of the tumour without residual disease plus chemotherapy and/or irradiation [15]. Galloway and Ridge in their study suggests that surgical resection followed by irradiation is an accepted treatment for CUP with only topical spread (T0N1). Nevertheless, this is an uncommon presentation because usually the disease is already spread in at least two lymph nodes. The identification of the primary location usually follows a period of time [4]. In these situation ESMO guidelines is more specific suggesting specific chemotherapy regimen according to the type of primary location [13,14]. However, in our case there is no evidence of lymph node tissue infiltration. Additionally, the uncommon location of CUP within the soft tissues of the supraclavicular region is rarely mentioned in the literature as well as the absence of lymph node infiltration like our case. Giordano et al. describe a case of adenocarcinoma of unknown primary in a patient’s finger. The tumor was located to a distal phalanx but no periostal reaction was observed. Histopathology of the lesion confirmed the adenocarcinoma of unknown primary [15]. On the other hand, cutaneous metastasis of CUP have been reported. Such cases are already metastatic with very low prognosis [16]. In contrast, the follow-up of our patient was free of disease which is extremely rare. This is questionable and there is no literature referral about management of this kind of location. From a surgical point of view the most common tumours in the supraclavicular region is either from Virchow lymph node, primary clavicle bone tumours or less frequently from secondary metastasis to the clavicle [17,18]. Surgical management in these cases requires a detailed surgical plan and a multidisplinary team approach. Important structures like branchial plexus, recurrent larungeal nerve (left side), thoracic duct (left side), subclavian artery, subclavian vein, innominate vein and lung parenchyma that must be protected during tumour recection [19]. Additionally, when a malignant lesion located in the neck region, like in our case, careful dissection of the common carotid artery, vagus nerve, internal jugular vein and thyroid gland may be required in the context of the good oncologic outcome [20,21].

In conclusion, CUP is a clinical entity most commonly presented as cervical adenopathy. Each patient should undergo a variety of diagnostic procedures in order to locate the tumour primary. In most of the cases the disease is already metastatic. In general, surgery plays a minor role in the management, however if applicable, it must be well organized. The approach must be multidisciplinary with the target of longer survival and better quality of life.

## Sources of funding

No funding of this study exists.

## Ethical approval

The case report is approved by the ethical committee of the hospital.

## Consent

The patient gave his consent for publication of his case.

The consent is available at any time is needed

## Author contribution

Georgios Geropoulos (manuscript preparation)

Sofoklis Mitsos (data analysis and manuscript preparation)

Lampridis Savvas (manuscript preparation)

Martin Hayward (study concept and data collection)

Marco Scarci (study concept and design, data collection)

Nikolaos Panagiotopoulos (study concept and design, data collection)

## Registration of research studies

NA.

## Guarantor

Georgios Geropoulos.

## Provenance and peer review

Not commissioned, externally peer-reviewed.

## Declaration of Competing Interest

No conflicts of interest of all authors.
